# Production of nouns and adjectives of children with cochlear implants and of children with typical hearing

**DOI:** 10.1016/j.heliyon.2023.e23496

**Published:** 2023-12-11

**Authors:** Cristina Cambra, Encarna Pérez, Josep-Maria Losilla

**Affiliations:** a Department of Basic, Developmental and Educational Psychology, Faculty of Psychology, Universitat Autònoma de Barcelona, 08193, Bellaterra, (Cerdanyola Del Vallès), Spain; b Department of Psychobiology and Health Sciences Methodology, Faculty of Psychology, Universitat Autònoma de Barcelona, 08193, Bellaterra, (Cerdanyola Del Vallès) , Spain

**Keywords:** Vocabulary, Nouns, Adjectives, Children, Cochlear implant

## Abstract

This analytical cross-sectional study aimed to investigate the production of nouns and adjectives in 62 children between the ages 5 and 7, with 31 children having Cochlear Implants (CIs) and 31 children having Typical Hearing (TH). The study compaired their performance in a picture naming test of nouns and adjectives. Poisson regression models were fitted to compare the responses of both groups of children, and intra-subject differences between responses to the noun and adjective naming tasks were also analyzed. The results showed that both groups of children produced the same number of non-responses of nouns and of adjectives and a higher number of correct productions of nouns than of adjectives. However, children with CIs produced more errors when naming adjectives than when naming nouns, while this difference is not observed in children with TH. The comparative analysis between both groups of children indicates that children with CIs produced a higher proportion of non-responses when naming nouns, but the same proportion as children with TH when naming adjectives. Children with CIs also produced fewer correct nouns and adjectives and more errors than children with TH. Vocabulary expansion and repair of production errors in children with CIs should be targeted by speech-language pathologists in intervention programs.

## 1 Introduction

The linguistic competence acquired by children after cochlear implantation has been studied in numerous investigations. Their results coincide in pointing out that the period after children receive the implant (which is known as hearing age) has a positive effect on the language acquisition process [[1], [2], [3], [4], [5], [6], [7], [8], [9], [10], [11], [12], [13]].

Despite this benefit, the review carried out by Lund [14] of the studies published between 1990 and 2015, exploring the receptive and expressive vocabulary of children with cochlear implants (CIs) and with typical hearing (TH), found divergent results. Some studies concluded that children with CIs achieved a level of vocabulary appropriate to their age [5,6,[15], [16], [17]]. Other studies, on the other hand, indicated that a vocabulary level comparable to that of their peers with TH was not achieved [7,[18], [19], [20], [21], [22]].

### 1.1 Production of nouns and adjectives of children with CIs

Le Normand, Ouellet & Cohen [23] carried out a longitudinal study to understand the spontaneous oral production of 17 children with CIs between 22 and 76 months of age during a symbolic play activity. The samples were collected every six months for three years and they determined that, two years after implantation, the total number of words produced (nouns, verbs and adjectives) was significantly lower than in the group of children with TH. However, three years after implantation, the children with CIs had made progress and the differences disappeared, even demonstrating a higher average production of adjectives than the control group.

Nouns constitute the first syntactic category acquired by children due to parental encouragement, as they often prompt children with the question “What is this?”. However, it has been found that parents infrequently ask “What is it like?” or “What's happening to it?” which would provoke the semantic production of adjectives [24]. Studies that compared children's production of both types of words showed a higher frequency of nouns than of adjectives [25,26]. Cutillas & Tolchinsky [27] observed an increase, with age, in the production of adjectives in narrative discourse, especially after 11 years of age.

Differences in the frequency of the use of nouns and adjectives has also been analyzed in some studies of children with hearing loss. The study by Välimaa et al. [28], which analyzed the vocabulary acquisition of deaf babies during the first year after cochlear implantation, pointed out that nouns are the most frequent semantic category in their vocabulary, while the percentage of adjectives is less than 10 %. Tribushinin et al. [29] conducted a longitudinal study to analyze the production of adjectives in 9 children with CIs who were followed up annually, from 2 to 7 years of age. They found no significant differences between the participants with CIs and the control group with TH in the frequency and diversity of adjectives used in a spontaneous communicative situation.

### 1.2 Difficulties in the production of nouns and adjectives by children with CIs

Lexical production difficulties have also been studied in children with CIs in a variety of tasks. A study by Boons et al. [30] carried out with 70 children with CIs and TH, between 5 and 13 years old, underscored the difficulties shown by deaf children in a task where they were asked to produce nouns, actions and categories with images from *The Expressive One Word Picture Vocabulary Test* [31]. They observed that the most frequent behaviour of children with CIs was not answering and they only exhibited significantly fewer errors than the group with TH of the type where an incorrect word from the same semantic field as the correct answer was given (semantic errors). The results of Wiig et al. [32], which explored the production of adjectives with the CELF language test, showed that only children with CIs who had a low morphosyntactic level made more production mistakes than the group with TH. Such differences were not observed when the level was suitable for their age.

The influence of the type of cochlear implant has also shown contradictory results. Guo, McGregor & Spencer [33] distributed the *MacArthur Communicative Development Inventories* [34] to 36 children with CIs (20 with unilateral CIs and 16 with bilateral CIs) and they found a correlation between the frequency with which the word appeared and the acquisition of nouns, verbs and adjectives after the implant when it was bilateral, but not when the implant was unilateral. In a more recent study, Cambra et al. [2] found that the type of implant did not significantly impact the number of correctly produced nouns.

There are comparative studies about the lexical acquisition of nouns and adjectives in the adult hearing population [[35], [36], [37], [38], [39]]. Nevertheless, there is no record of any studies in children, with or without TH, which compare both lexical productions (nouns and adjectives) in the same task.

The discrepant results of the studies into the acquisition of lexicon of children with CIs, and the dearth of investigations on noun production and, even more so, on adjective production make it necessary to continue studying both lexical categories in more detail.

The objective of this study, therefore, is to explore the production of nouns and adjectives of children with CIs in a picture naming activity which will shed light on, first of all, whether children produce equal quantities of nouns and adjectives and whether such production differs between children with CIs and children with TH; secondly, whether the number of correct productions of nouns and adjectives is the same and whether this production differs between children with CIs and with TH; and thirdly, whether the profile of errors in the production of nouns and adjectives is similar and whether there are differences in the type of errors produced by the groups of participants.

The hypothesis of the study, based on the results of previous studies which compare the production of nouns and adjectives of children with CIs and that of children with TH, are presented hereunder.

H1. *Differences are expected between the number of non-responses of nouns and adjectives in children with CIs and in children with TH*. According to the results of the study by Boons et al. [30] children with CIs are expected to give more non-responses than children with TH when a noun is required. In other words, children with CIs will produce a lower number of nouns than children with TH. There are no studies that support the existence of differences between both groups in the quantity of non-responses when an adjective is requested.

H2 *It is hypothesised that the children with CIs will differ from children with TH in regards to correct nouns produced, but not adjectives*. As indicated by the study carried out by Davidson, Geers, & Nicholas [18] children with CI are expected to produce a lower number of correct nouns than children with TH. Nonetheless, from the studies of Le Normand, Ouellet & Cohen [23] and of Tribushinina et al. [29], no differences in the number of correct adjectives produced are expected to be found.

H3. *It is hypothesised that children with CIs and children with TH will produce the same types of errors at the same frequencies*. Based on the studies by Boons et al. [30] and Wiig et al. [32], it is expected that children with CIs and children with TH will show the same quantity of errors and the same profile of error production, both for nouns and for adjectives.

## 2 Methods

The study design was analytical and cross-sectional, comparing the results obtained by two representative groups of children, one with CIs and one with TH, in a picture naming task designed to test production of nouns and adjectives.

### 2.1 Participants

The study sample was composed of 62 children, 31 with CIs (18 girls and 13 boys) and 31 with TH (18 girls and 13 boys), between 5 and 7 years old, who were in Early Childhood Education (P5) or in the first year of Primary Education in inclusive schools in Catalonia. All of them were children of hearing parents and used spoken Catalan language. The participants were selected through speech-language pathologists who are part of the team of professionals at the Educational Resource Centres for Children with Hearing Loss (CREDA) of the *Departament d’Ensenyament de la Generalitat de Catalunya*, responsible for carrying out speech-language therapy intervention for students with hearing loss and for responding to their educational needs. In Catalonia there are 10 CREDAs and all of them were part of the sample. An informative meeting was held with all the directors of the CREDAs to present the objective of the study and obtain their consent to collect the data from the students with hearing loss who met the requirements established in the study to be part of the sample.

The selection criteria for the group with CIs were prelingual deafness (from birth or before language acquisition); unilateral or bilateral cochlear implant for two or more years (hearing age equal to or greater than two years) and absence of associated disability. A strength of the study is that the sample represents the entire population of Catalonia that meets these selection criteria (Table 1).

**Table 1:** Statistical description of the sample.

	Children with Typical Hearing (TH)	Children with cochlear implants (CIs)	Total sample
	n (%)	n (%)	n (%)
Gender			
Male	13 (42)	13 (42)	26 (58)
Female	18 (58)	18 (58)	36 (58)
Type of implant			
Unilateral		21 (67.7)	
Bilateral		10 (32.3)	
	Average (SD)	Average (SD)	Average (SD)
Chronological age (years)	6.03 (0.71)	5.97 (0.75)	6.0 (0.72)
Age at implantation (months)		26.58 (15.04)	
Hearing age (months)		52.03 (15.15)	

For each child with CI, an age- and sex-matched peer with typical hearing was selected from their class group with a similar academic level and socioeconomic background. These were controlled for by comparing academic grades and inferring parental salary based on their profession.

The study has the approval of the Ethics Committee for Human and Animal Experimentation (CEEAH) of the Universitat Autònoma de Barcelona (CEEAH 5810) which imposes the obligation to inform families about the purpose of the study and request their consent for their children's participation.

## 3 Materials

All 28 images from the Speech Retardation Analysis test (A-RE-PA) [40] were used. The images represent food, animals, objects, musical instruments, transportation, and professions to prompt the production of nouns. The test was prepared by the authors for the phonological study of children from 3 years of age. This ensures that the images are suitable for the age range in our study. In addition to these 28 nouns, another 13 nouns and 10 complementary adjectives were selected, also represented in the images, which were requested only when children correctly produced the noun which these nouns and adjectives complemented. For example, when responding with the noun “queen”, they were asked to produce a supplementary noun “crown” and an adjective that would describe the queen. Therefore, the number of words requested was between a minimum of 28 and a maximum of 51. Table 2 lists the nouns and adjectives used in the test.

**Table 2:** List of nouns from the AREPA test, with complementary nouns and adjectives used in the study.

Nouns (AREPA)	Complementary nouns	Adjectives
1. Box		2.Open
3. Yogurt		4.Closed/Full
5. Football player		
6. Helmet		
7. Queen	8. Crown	9.Big/Tall
10. Helicopter	11. Propeller	
12. Car		
13. Tortoise	14. Shell	15. Hard
16. Moon		
17. Soup	18. Spoon	
19. Fairy	20. Wand	
21. Bus		
22. Droplet		
23. Fish		
24. Carrots		25. Orange
26. Pear		
27. Drum		
28. Horn		
29. Chocolate		30.Dark/Sweet
31. Pitcher	32. Handle	33.Empty
34. Star		
35. Meat	36. Peppers	
37. Elephant	38. Trunk	
40. Sheep	39. Tusks	
43. Melon	41. Baby	42. Small
46. Garage	47. House	44. Divided 45. Whole
48. Doctor	49. Glasses	
51. Doorbell	50. White coat	

### 3.1 Procedure

The test was conducted in a room in each participant's school. The images were shown one by one by speech-language pathologists who were experienced in administering the tests to children with language disorders. They were asked “What is it?” if a noun was requested, or “What is it like?/How does it feel?” if an adjective was required. Following the same criteria applied by Wechsler-Kashi et al. [41], once the image was shown, the child was given 4 s to respond. If after this time an answer was not obtained, the image was removed and the next one was shown.

The responses were recorded and were later transcribed by two judges (judge 1: NM; judge 2: CC) and reviewed by a third judge (judge 3: IE). The judges received two training sessions before performing the codification of answers: in the first session, the analysis categories were explained in detail with examples, and in the second session, the judges practiced codifying transcripts of cases unrelated to the study, in order to resolve doubts. The responses were coded following the categories collected in the *Protocolo de Análisis de las Dificultades del Lenguaje* PADIL [42]. The judges made their evaluations independently and resolved the few discrepancies in data coding by consensus.

### 3.2 Measures and categories of analysis

The hearing condition of all participants was registered (CI, TH), as well as the sex (male, female), the chronological age (in months) and the hearing age (in months) (Table 1).

Categories of analysis and quantitative variables were established for each of the lexical categories studied, nouns and adjectives, which are shown in Table 3.

**Table 3:** Categories of analysis and quantitative variables.

Category	Quantitative Variables
**Non-responses**	Number of words in which no response is obtained.
**Correct responses**	Number of suitable words.
**Errors**	Total number of words with lexical errors
Semantic	Number of erroneous responses in which a word from the correct semantic field is produced
Morphologic	Number of errors due to incorrect use of derivational morphology.
Functional Definition	Number of responses that define the function of the word instead of producing the word.
Circumlocution	Number of responses in which a phrase is produced instead of the word.
Imprecise Lexicon	Number of erroneous words that are deictic terms or generic words.
Part-for-whole	Number words that represent only a part of the requested word.
Perceptive	Number of erroneous words due to interference of the perceptive aspect of the image.
Unrelated Lexicon	Number of words that have no relation with the requested word
Inexistent Lexicon	Number of words that do not exist

### 3.3 Data analysis

Considering the measurement scale of the outcomes registered (counts/number of instances) and the non-experimental nature of our study design, Poisson regression models were fitted to investigate the responses of children with CIs and children with TH, including gender and age as control variables [[43], [44], [45]]. The Incidence Rate Ratio (IRR) was calculated as the measure of relative risk, as well as its 95 % confidence interval (95 % CI) and *p*-values.

The exact probabilities of the intra-subject differences between the response ratios obtained in the picture naming tasks of nouns and adjectives were also calculated using the Wilcoxon signed-rank test for paired differences of repeated measurements on a single sample [46].

Statistical tests were considered significant at P < 0.05. All statistics were processed with Stata/SE v16 (StataCorp, 2019).

## 4 Results

**Fig. 1:**
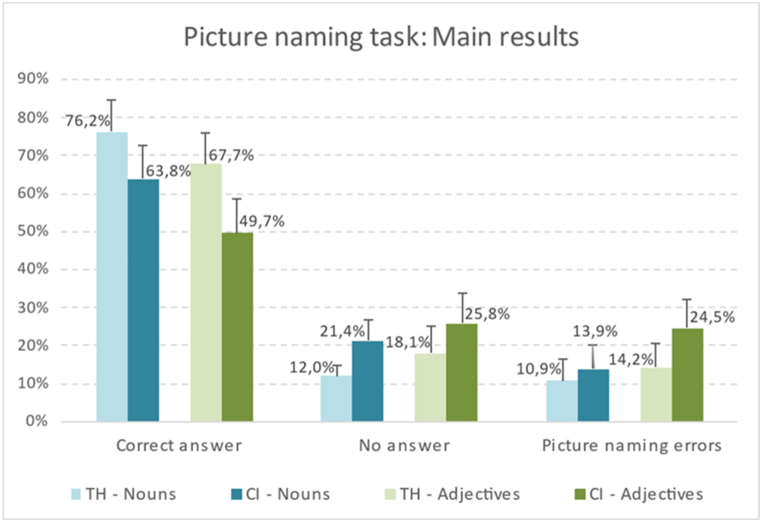
Percentage distribution of the main results in the picture naming task of nouns and adjectives for the students with cochlear implants (CIs) and for students with typical hearing (TH).

**Fig. 2:**
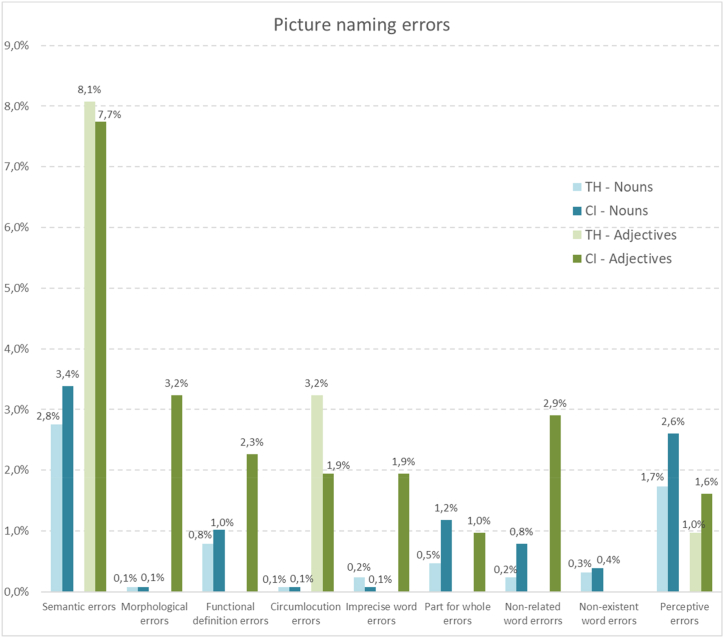
Percentage distribution of the types of errors in the picture naming task of nouns and adjectives of the students with cochlear implants (CIs) and the students with typical hearing (TH).

Here below, the results of the analysis are shown, according to the objectives and hypotheses formulated. Table 1 presents the characteristics of the participants with CIs and the participants with TH. Table 4 contains the statistical analysis of the differences obtained by each group of the sample in the production of nouns and adjectives. Table 5 shows the comparative analysis of the results obtained by the children with CIs and the children with TH, which correspond to the hypotheses H1, H2, and H3. Fig. 1 shows the percentage distribution of the principal results in the picture naming task of nouns and adjectives of the children with CIs and the children with TH and Fig. 2 shows the profile of production errors of nouns and adjectives of the children with CIs and the children with TH.

**Table 4:** Differences between the responses obtained in the noun and adjective naming tasks.

	Children with cochlear implants (CIs)						Children with typical hearing (TH)					
	Noun %	Adj. %	+	–	=	P	Noun %	Adj. %	+	–	=	P
No answer	21.40	25.81	14	17	0	0.375	12.04	18.07	13	17	1	0.117
Correct answer	63.81	49.68	24	7	0	<0.001*	76.24	67.74	20	11	0	0.043*
Total errors	13.93	24.52	8	23	0	<0.001*	10.86	14.19	9	22	0	0.075
Semantic errors	3.38	7.74	10	15	6	0.047*	2.75	8.07	11	17	3	0.006*
Morphological errors	0.08	3.23	1	10	20	0.002*	0.08	0.00	1	0	30	1.0
Functional definition errors	1.02	2.26	10	6	15	0.735	0.79	0.00	7	0	24	0.016*
Circumlocution errors	0.08	1.94	0	5	26	0.0625	0.08	3.23	1	8	22	0.008*
Imprecise lexicon errors	0.08	1.94	0	6	25	0.031*	0.24	0.00	3	0	28	0.250
Part-for-whole errors	1.18	0.97	12	3	16	0.058	0.47	0.00	6	0	25	0.031*
Unrelated lexicon errors	0.79	2.90	7	7	17	0.617	0.24	0.00	3	0	28	0.250
Inexistent word errors	0.39	0.00	3	0	28	0.250	0.32	0.00	4	0	27	0.125
Perceptive errors	2.60	1.61	18	5	8	0.092	1.73	0.97	12	3	16	0.043*

*Note*: % Noun: percentage obtained in the noun naming task; % Adj.: percentage obtained in the adjective naming task; +: higher % in denomination of nouns than of adjectives; –: lower % in denomination of nouns than of adjectives; = : same % in denomination of nouns as of adjectives; P: Exact probability in the Wilcoxon signed-rank test for paired differences (Noun – Adj.); *: Statistically significant (P < 0.05).

**Table 5:** Comparative analysis between children with cochlear implants (CIs) and children with typical hearing (TH; reference group) in the picture naming task.

Reference group: children with typical hearing (TH)	Noun picture naming task		Adjective picture naming task	
	IRR_adj_ (95 % CI)	P	IRR_adj_ (95 % CI)	P
No answer	1.778 (1.458–2.168)	<.001*	1.390 (0.987–1.957)	.060
Correct answer	0.838 (0.763–0.920)	<.001*	0.738 (0.599–0.909)	.004*
Total errors	1.270 (1.016–1.587)	0.036*	1.733 (1.195–2.512)	.004*
Semantic errors	1.204 (0.770–1.883)	0.415	0.966 (0.552–1.692)	0.904
Morphological errors	1.002 (0.063–16.043)	0.999	n.c	
Functional definition errors	1.292 (0.565–2.952)	0.544	n.c.	
Circumlocution errors	1.002 (0.063–16.043)	0.999	0.606 (0.220–1.667)	0.332
Imprecise word errors	0.326 (0.034–3.161)	0.334	n.c.	
Part-for-whole errors	2.309 (0.892–5.977)	0.085	n.c.	
Unrelated word errors	2.873 (0.783–10.542)	0.112	n.c.	
Inexistent word errors	1.253 (0.336–4.668)	0.736	–	–
Perceptive errors	1.500 (0.875–2.574)	0.141	1.685 (0.401–7.082)	0.476

*Note:* Adjusted Poisson regression coefficients (IRR_adj_), 95 % confidence intervals (95 % CI) and p-values (P). All regression models include sex and age as adjustment variables. * IRR_adj_ statistically significant (P < 0.05). --: zero in all observations; n.c.: convergence not achieved.

Quantity of non-responses produced by the children with CIs and by the children with TH in the picture naming task, and comparison between the two groups.

No statistically significant differences are observed between the number of non-responses in the naming task of nouns and adjectives either in the children with CIs or in the children with TH (Table 4).

However, the analysis of the non-responses for the requested word has revealed statistically significant differences between the students with CIs and the students with TH when a noun was requested (Table 5; Fig. 1). The students with CIs failed to respond between 1.5 and 2.2 times more than the students with TH. This trend is also observed with respect to the non-responses when adjectives are requested, even though the difference between children with CIs and children with TH is not statistically significant in this case.

Correct production of nouns and adjectives of children with CIs and children with TH, and comparison between the two groups.

The number of correct productions of nouns was significantly superior to that of adjectives, both in the case of children with CIs and children with TH (Table 4). Children with CIs produced 63.81 % of the correct nouns and 49.68 % of the adjectives (*p* < 0.001); while children with TH produced 76.24 % of the correct nouns and 67.74 % of the adjectives (*p* < 0.05).

Statistically significant differences have also been observed between the two groups of children in the correct production of nouns and adjectives (Table 5; Fig. 1). Children with CIs produced between 8 % and 23.72 % less correct nouns than the group with TH (IRR_adj_ = 0.838; 95 % CI: 0.763–0.920) and between 9.1 % and 40.1 % less correct adjectives than the group with TH (IRR_adj_ = 0.738; 95 % CI: 0.599–0.909).

### 4.1 Errors of the children with CIs and the children with TH in the production of nouns and adjectives, and differences between the two groups

As shown in Table 4, the quantity of errors that the children with CIs made with adjectives (24.52 %) is significantly greater than those with nouns (13.93 %; *p* < 0.001*).* In contrast, even though the trend is the same as for the group of children with CIs, in the group of children with TH no statistically significant differences are observed in the quantity of errors for nouns (10.86 %) and adjectives (14.19 %).

Regarding the type of errors, both children with CIs and children with TH made a significantly larger number of semantic errors naming adjectives than naming nouns (*p* < 0.05). In addition to this type of error, children with CIs also showed a significantly greater number of morphological errors (*p* = 0.002) and of imprecise word errors (*p* = 0.031) when they produced adjectives than when they produced nouns. In contrast, children with TH showed a significantly larger percentage of circumlocutions when producing adjectives than when producing nouns (*p* = 0.008) and a significantly larger percentage of functional definition errors (*p* = 0.016), part-of-the-whole errors (*p* = 0.031) and of the perceptive type (*p* = 0.043) when they were prompted to produce a noun than when they were prompted to produce an adjective.

The analysis of the errors also shows statistically significant differences between the two groups in the total quantity of errors of nouns and adjectives (Table 5; Fig. 1). Children with CIs produced between 1.6 % and 58.7 % more total errors in the production of nouns than the group with TH (IRR_adj_ = 1.270; 95%CI: 1.016–1.587) and between 19.5 % and 151.2 % more total errors in the production of adjectives than the group with TH (IRR_adj_ = 1.733; 95 % CI: 1.195–2.512).

Analysis of the types of errors made by the children revealed that the group with CIs made an equal or greater number of errors than the group with TH across all error types when producing nouns, although these differences are not statistically significant (Fig. 2). The only contrasting category is the case of the use of imprecise lexicon, where the total number of errors for nouns of the group with TH is slightly higher than that of the group with CIs (0.1 % in the students with CIs vs 0.2 % in the students with TH) without being statistically significant.

In the production of adjectives, taking into account the error types with a sufficient number of responses to perform the statistical analysis, the percentage of perceptual errors of students with CIs is slightly higher than that produced by students with TH (1.6 % vs. 1 %), contrary to the percentage of semantic errors (7.7 % vs. 8.1 %) and circumlocutions (1.9 % vs. 3.2 %) (Fig. 2), although none of these differences reach statistical significance.

## 5 Discussion

The aim of this study was to deepen the understanding of the lexical production of nouns and adjectives of students with CIs and TH by means of a picture naming activity. The quantity of non-responses, correct responses and the quantity and type of errors were analyzed and compared between the two groups.

The results showed that children between 5 and 7 years of age with cochlear implants produced the same number of non-responses when prompted to produce nouns as when prompted to produce adjectives, a quantity of correct nouns significantly greater than that of adjectives, and a greater quantity of errors when naming adjectives as opposed to nouns, with significantly more semantic and morphologic errors as well as errors of imprecise lexicon.

In the case of the children with TH, they mimic the children with CIs in the fact that they produce the same number of non-responses for nouns as for adjectives and achieve a higher number of correct nouns than adjectives. Regarding the erroneous responses, no differences were observed in the number of errors when producing nouns or adjectives, but among the types of errors, circumlocutions and semantic errors were significantly more common when producing adjectives whereas errors by functional definition, part-for-whole errors and perceptive errors were significantly more frequent for nouns.

Comparison of the production of nouns and adjectives of the two groups revealed that the children with CIs failed to respond to a greater number of prompts to produce nouns, while exhibiting the same proportion of non-responses for adjectives as the group with TH. They produced fewer correct nouns and adjectives than the children with TH and committed more errors. However, the typology of errors produced for nouns and adjectives did not significantly differ between the two groups of children.

This study shows, therefore, that the behaviour of the two groups, the children with CIs and the children with TH, did not differ in the number of non-responses of nouns and adjectives, nor in the superior number of correct productions of nouns versus adjectives in the picture naming activity. According to Serra et al. [24], nouns constitute the broadest category of the repertoire of words acquired by children, a fact that justifies that the number of correct productions of nouns was greater than that of adjectives, since this semantic category does not appear as frequently in their vocabulary [25,26,28].

Regarding the errors produced, children with CIs produced more errors for adjectives than for nouns, unlike children with TH in which errors were equally distributed between the semantic categories. This result partially concurs with that found by Wiig et al. [32] who only found a greater number of errors in the production of adjectives in the CELF language test when children with CIs had a language level lower than that corresponding to their age. This same study highlighted morphological errors as one of the most frequent types of errors in children with CIs when producing adjectives, as occurred in the present study. Likewise, the results of this study indicate that the most common type of error by both children with CIs and children with TH was semantic when a word from the same semantic field as the correct answer is produced. According to Silvestre & Solé [47], the ability to organize words of the same semantic field is a cognitive-linguistic ability that develops between 4 and 6 years of age, so at these ages it can lead to errors, as has been observed in the children in this study.

Comparative analysis of the responses of children with CIs and children with TH shows that the number of non-responses of nouns of children with CI was higher than that of children with TH. Therefore, the hypothesis proposed in the study regarding the higher number of non-responses is supported (H1), coinciding with the results of the study by Boons et al. [30]. In contrast, no differences were found in the number of adjective non-responses between children with CIs and children with TH. Therefore, even though children are exposed to a greater number of nouns than adjectives, as pointed out by Serra et al. [24], this does not guarantee, in the case of children with CIs, their automatic acquisition. One of the practical implications that derive from these results is the need to consider the expansion of the lexical repertoire of students with CIs in the design of language intervention programs. In this sense, the focus should not be directed solely toward the acquisition of a greater number of adjectives, but also of nouns, due to the observation that children with CIs fail to respond more frequently than children with TH when asked to produce nouns. The differences between the vocabulary acquired by children with CIs and that of children with TH have already been pointed out in previous studies collected in the review by Lund [14].

The results have also shown that the number of correct nouns and adjectives of children with CIs, as mentioned in H2, is significantly lower than that of children with TH, and the number of errors is significantly higher (H3), unlike the results of Wiig et al. [32] who found no significant differences in the production of adjectives when children with CIs had the same morphosyntactic level as children with TH. The practical implications of these results have to do with the need to focus linguistic intervention on two aspects: on the one hand, expanding the vocabulary of children with CIs and, on the other hand, reducing the number of errors they make. In this sense, analysis of the types of errors that children with CIs produce will allow speech-language pathologists to focus specifically on more common types of errors.

In the noun production task, both the group of children with CIs and those with TH make every type of error described, but no significant differences were observed in their frequency. In relation to the types of errors in the production of adjectives, although children with TH do not exhibit all the typology described, no differences were observed between the two groups in the frequency of errors. Therefore, the hypothesis formulated in this study regarding the non-existence of a specific error typology profile for children with CIs is supported (H3), which agrees with the studies by Boons et al. [30] and Wiig et al. [32].

In conclusion, the results of this study showed that children with CIs and children with TH produced the same number of non-responses of nouns and of adjectives and a higher number of correct productions of nouns than of adjectives. However, children with CIs produced more errors when naming adjectives than when naming nouns, while this difference is not observed in children with TH. Comparative analysis of the responses of children with CIs and children with TH indicates that the number of non-responses of nouns of children with CI was higher than that of children with TH. In contrast, no differences were found in the number of adjective non-responses between the two groups of children.

The difficulties in the production of nouns and adjectives in children with CIs should be considered by speech-language pathologists. Future research in other areas of language, such as pragmatics or morphosyntax, is required to complete the linguistic profile of children with CIs and to improve intervention programs.

## 6 Limitations of the study

One of the limitations of this study is the fact that it is a cross-sectional study. It would be desirable to carry out a longitudinal study that would make it possible to assess the lexical development of children as they age and to be able to assess the process of lexical acquisition of children with CIs as a result of the linguistic intervention carried out. However, one strength of this study is the fact that, although the sample is small, it was carried out with a representative sample of children with CIs, from 5 to 7 years of age, which constitutes the entire population of this age group in Catalonia that meets the inclusion criteria established in the study.

A second limitation of the study is the fact that it was not possible to calculate the degree of agreement between the 3 judges who independently carried out the coding of the children's responses, due to the fact that only the final codification was recorded, resulting from their consensus once the few discrepancies that occurred were resolved.

## Funding

This work was supported by the Spanish 10.13039/100014440Ministry of Science, Innovation and Universities (grant number PID2022-141403NB-I00 funded by MCIN/10.13039/100007622AEI/10.13039/501100011033/FEDER, 10.13039/501100012637UE).

## Data availability statement

Data associated with this study has not been deposited into a publicly available repository.

Data will be made available on request.

## CRediT authorship contribution statement

**Cristina Cambra:** Writing - review & editing, Writing - original draft, Methodology, Investigation, Formal analysis, Data curation, Conceptualization. **Encarna Pérez:** Writing - review & editing, Writing - original draft, Methodology, Investigation, Formal analysis, Data curation, Conceptualization. **Josep-Maria Losilla:** Writing - review & editing, Writing - original draft, Methodology, Formal analysis.

## Declaration of competing interest

The autors declare no conflicte of interest.
